# Evaluation of PSII Thermal Stability in Grapevine Germplasm and Identification of Candidate Genes Based on a Genome-Wide Association Study

**DOI:** 10.3390/ijms27209214

**Published:** 2026-10-17

**Authors:** Wentong Wu, Lei Sun, Rui Xu, Rongrong Guo, Jingyang Wang, Chenyang Hu, Xiucai Fan

**Affiliations:** 1 National Key Laboratory for Germplasm Innovation & Utilization of Horticultural Crops, Zhengzhou Fruit Research Institute, Chinese Academy of Agricultural Sciences, Zhengzhou 450009, China; wuwentong26@163.com (W.W.); sunlei01@caas.cn (L.S.); xurui@caas.cn (R.X.); wjyang1104@163.com (J.W.); hcy9983mu@163.com (C.H.); 2 Chuxiong Yunguo Agriculture Technology Research Institute, Chuxiong 675000, China; 3 Guangxi Academy of Agricultural Sciences, Nanning 530007, China; guorongrong3434@163.com

**Keywords:** diplotype, Fv/Fm, genome-wide association study, grapevine germplasm, PSII thermal stability

## Abstract

Heat stress damages the photosynthetic apparatus of grapevine leaves. It also restricts plant growth and reduces fruit yield and quality. However, natural variation in photosystem II (PSII) thermal stability under heat stress in grapevine and its genetic basis remain poorly understood. Here, we evaluated 404 genetically diverse grapevine accessions. We measured the maximum quantum efficiency of photosystem II (Fv/Fm) after treatment at 47 °C. Phenotypic analysis, cluster analysis and genome-wide association analysis were then used to investigate the genetic basis of PSII thermal stability. The coefficient of variation for Fv/Fm increased from 4.52% at 25 °C to 40.60% at 47 °C. Wild accessions generally retained higher Fv/Fm than interspecific hybrids and *Vitis vinifera* cultivars. We also identified 21 accessions with relatively high PSII thermal stability. A genome-wide association analysis of 5,074,860 high-quality SNPs detected a suggestive association signal on chromosome 4. The lead SNP was Chr4_1743280_G_A. We integrated gene annotations within the associated interval, putatively functional variants and diplotype–phenotype comparisons. This analysis prioritized seven candidate genes potentially associated with variation in PSII thermal stability. The Hap2/Hap2 diplotype of *Vitvi005414* was associated with higher Fv/Fm. However, these associations should be regarded as candidate hypotheses for further validation rather than evidence of causal relationships. These findings provide candidate loci, genes and diplotypes for understanding the genetic basis of PSII thermal stability. They also support the use of germplasm with relatively high PSII thermal stability, validation of candidate variants and future breeding for improved heat resilience.

## 1. Introduction

Grapevine is a perennial fruit crop of major economic importance and is widely cultivated worldwide for fresh consumption, winemaking, raisin production and other processed products. According to the International Organisation of Vine and Wine (OIV), the global vineyard surface area was 7.0 million ha in 2025 [1]. With the increasing frequency and intensity of extreme heat events under climate change, heat stress can impair grapevine growth and photosynthesis, disrupt fruit development, and reduce yield and quality, thereby becoming an important constraint on sustainable grape production [2]. Characterizing variation in grapevine heat responses and its genetic basis is therefore important for identifying useful germplasm and supporting genetic improvement.

The photosynthetic apparatus, particularly photosystem II (PSII), is highly sensitive to elevated temperature. Heat stress can disrupt PSII activity, thylakoid membrane stability and photosynthetic electron transport, thereby reducing photosynthetic efficiency [3]. Chlorophyll fluorescence provides a rapid and non-destructive assessment of PSII function, and the maximum quantum efficiency of PSII (Fv/Fm) is widely used to evaluate photoinhibition and PSII damage [4]. In grapevine, heat treatment can markedly affect PSII photochemical efficiency, and Fv/Fm has been used as a useful phenotype for evaluating heat responses and PSII thermal stability [5,6].

Previous studies have used Fv/Fm and related physiological indicators to evaluate variation in heat responses among grapevine germplasm, including cultivars, interspecific hybrids and wild accessions [6,7,8,9]. Together, these studies support the use of Fv/Fm for rapid screening of variation in PSII thermal stability. However, most have focused on specific germplasm groups or phenotypic classification, whereas broad panels comprising genetically diverse *Vitis vinifera* cultivars, interspecific hybrids and wild accessions remain insufficiently evaluated under standardized conditions. Moreover, the genetic basis of natural variation in PSII thermal stability remains poorly resolved.

Grapevine heat responses are complex and involve multiple regulatory processes, including heat-shock responses, antioxidant defence, signalling and transcriptional regulation [10,11,12,13]. Recent studies have identified several regulators and natural variants involved in grapevine heat responses, including *HSFA2*, HSFB1 and miPEP398b [14,15,16]. However, these studies have mainly focused on individual regulators or specific genetic materials and do not fully explain the genetic architecture of PSII thermal stability in genetically diverse grapevine populations.

Genome-wide association studies (GWAS) provide a population-based approach for dissecting the genetic architecture of complex quantitative traits [17]. In grapevine, GWAS has been widely applied to diverse agronomic and fruit-quality traits, including berry weight, flesh texture, flavour and aroma, berry shape and postharvest traits [18,19,20,21,22]. More recently, GWAS combined with transcriptomic analysis identified *TTC4* as a regulator associated with natural variation in grapevine thermotolerance [23]. However, few studies have systematically investigated genomic loci, candidate variants and haplotype effects associated with Fv/Fm-based PSII thermal stability across a broad grapevine population.

Here, we evaluated heat responses in 404 grapevine accessions with diverse genetic backgrounds. Fv/Fm was measured after treatments at 25 °C and 47 °C to assess PSII thermal stability. We examined phenotypic variation, differences among germplasm groups and clustering patterns after treatment at 47 °C. Representative accessions with high or low PSII thermal stability were then identified. We further used Fv/Fm after treatment at 47 °C as the core phenotype for GWAS based on high-quality genome-wide SNPs. Association signals were integrated with gene annotations, putatively functional variants, haplotypes and diplotype–phenotype comparisons. This approach prioritized candidate genes associated with natural variation in PSII thermal stability and candidate diplotypes associated with higher Fv/Fm. The study provides a phenotypic basis for identifying grapevine germplasm with contrasting PSII thermal stability. It also provides genetic targets for functional validation and the development of molecular markers for future breeding aimed at improving heat resilience.

## 2. Results

### 2.1. Population Structure and Phenotypic Analysis

**Figure 1:**
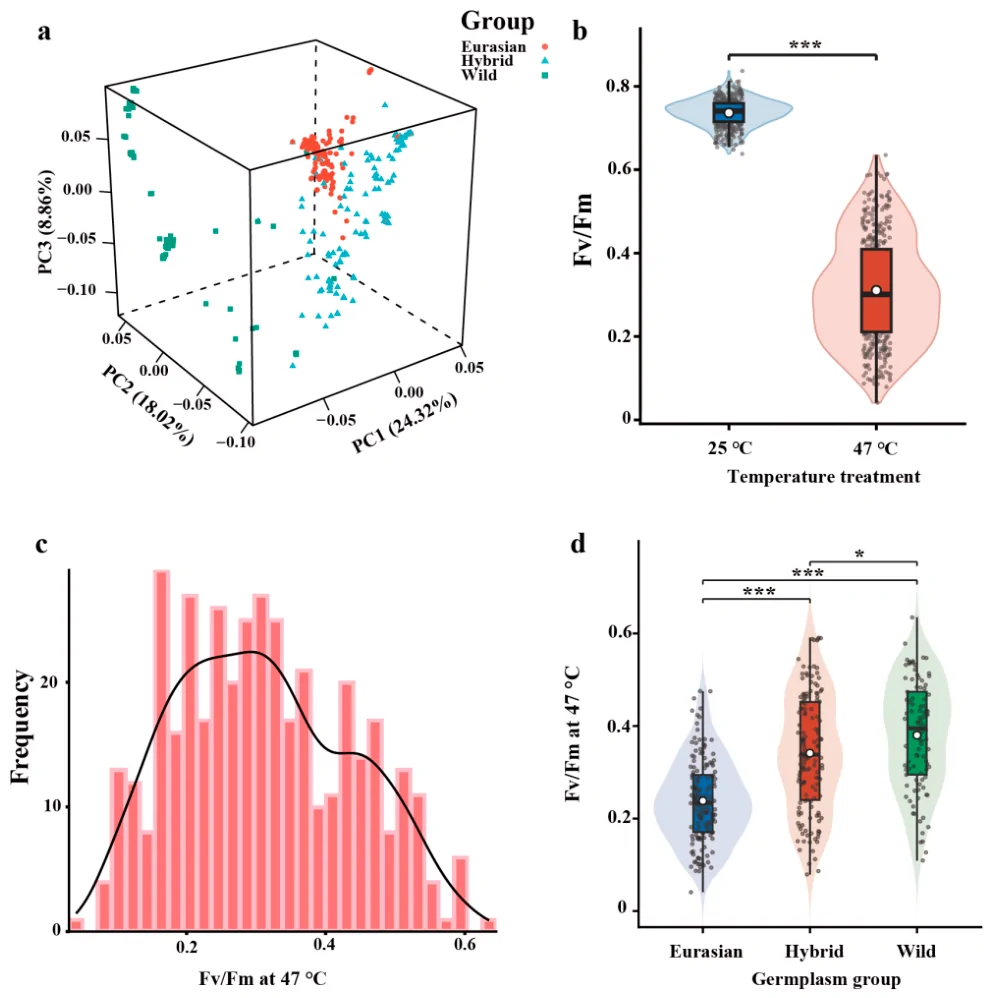

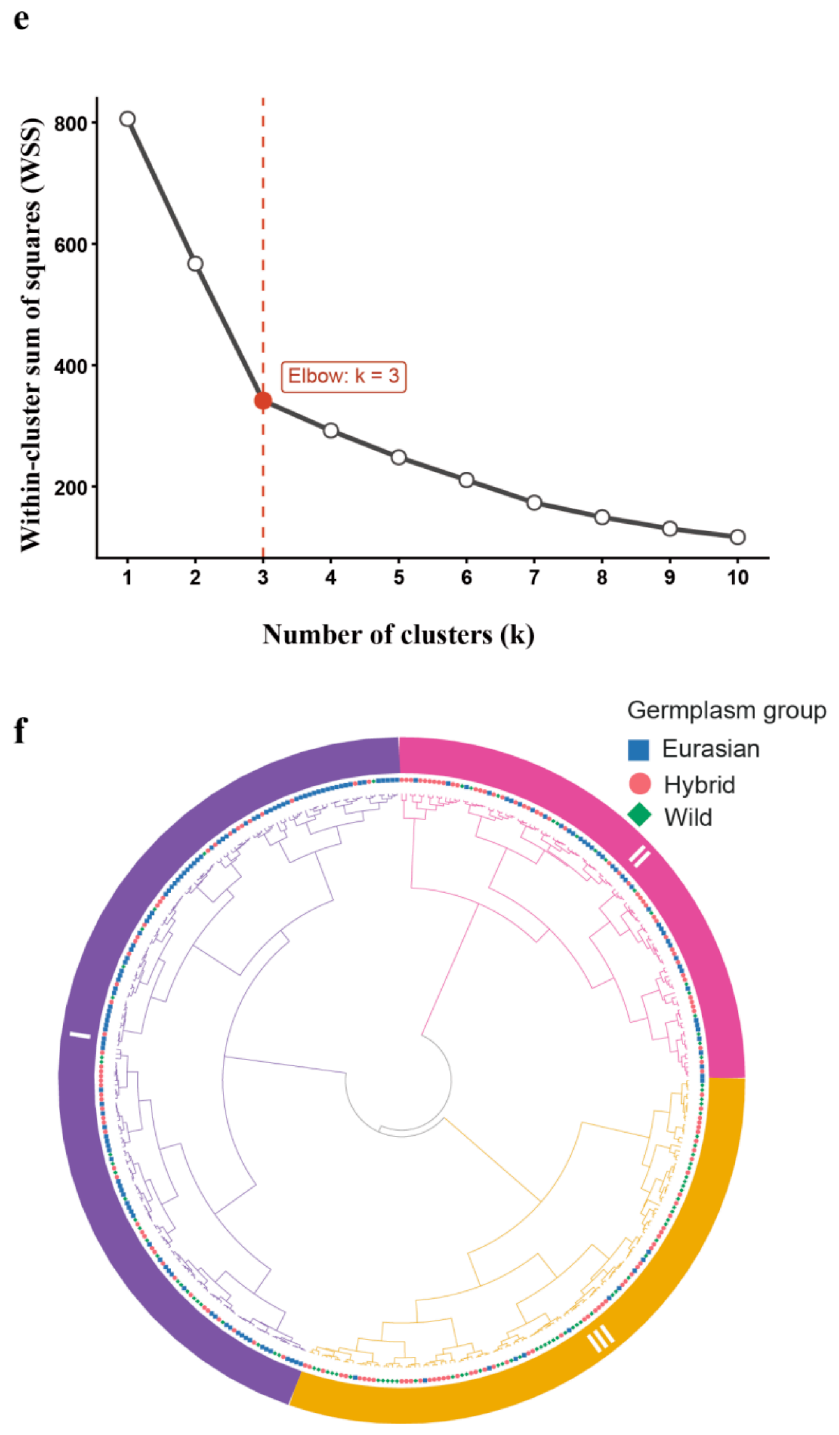
Population structure and variation in Fv/Fm-based PSII thermal stability among 404 grapevine accessions. (**a**) Three-dimensional principal component analysis based on genome-wide SNP data. (**b**) Distribution and paired comparison of Fv/Fm under the 25 °C and 47 °C treatments. (**c**) Frequency distribution of Fv/Fm after treatment at 47 °C. (**d**) Comparison of Fv/Fm after treatment at 47 °C among *Vitis vinifera* cultivars, interspecific hybrids and wild accessions. (**e**) Elbow plot for determining the number of clusters based on the within-cluster sum of squares. (**f**) Circular hierarchical clustering dendrogram generated using the Ward.D2 method and Fv/Fm at 47 °C. Clusters I–III are ordered by increasing mean Fv/Fm after treatment at 47 °C. Colours and symbols indicate different germplasm groups. * and *** indicate *p* < 0.05 and *p* < 0.001, respectively.

Principal component analysis (PCA) was performed for 404 grapevine accessions using genome-wide SNP data. The panel comprised 158 *V. vinifera* cultivars, 149 interspecific hybrids and 97 wild accessions. PC1, PC2 and PC3 explained 24.32%, 18.02% and 8.86% of the genetic variation, respectively, accounting for 51.20% in total. The three germplasm groups showed partial separation and some overlap in the principal component space, indicating detectable population structure. The principal components and kinship matrix were therefore included in the subsequent GWAS model to control for potential confounding caused by population structure and relatedness (Figure 1a).

Under the 25 °C control condition, Fv/Fm ranged from 0.638 to 0.837 across the 404 accessions, with a mean of 0.736, a standard deviation of 0.033 and a coefficient of variation of 4.52% (Figure 1b and Supplementary Table S1a). After treatment at 47 °C, Fv/Fm ranged from 0.041 to 0.635, with a mean of 0.310 and a standard deviation of 0.126. The coefficient of variation increased to 40.60%, and the phenotype showed a continuous distribution (Figure 1c and Supplementary Table S1a). All 404 accessions had lower Fv/Fm values at 47 °C than under the corresponding control condition. A paired-samples t-test showed a significant difference between the two temperature treatments (t = 67.14, *p* < 0.001; Figure 1b and Supplementary Table S1b).

The accessions were ranked according to their Fv/Fm values after treatment at 47 °C. The upper and lower 5% of the distribution were used to identify contrasting phenotypic extremes within the association panel. The top 5%, comprising 21 accessions with Fv/Fm ≥ 0.529, were designated as candidate accessions with relatively high PSII thermal stability, whereas the bottom 5%, comprising 21 accessions with Fv/Fm ≤ 0.115, were designated as candidate accessions with relatively low PSII thermal stability (Supplementary Table S2). This percentile-based screening was intended to identify relative phenotypic extremes rather than to define absolute categories of grapevine heat tolerance.

The five accessions with the highest Fv/Fm values were ‘Gaoshan No. 1’, ‘Beni Zuiho’, ‘Dulumei’, ‘Fenghou’ and ‘Red Queen’. The five accessions with the lowest values were ‘Gongyi Wuhebai’, ‘Yemeiren’, ‘Baisainie’, ‘Juyou’ and ‘Jade Seedless’.

Fv/Fm after treatment at 47 °C differed significantly among the three germplasm groups (F = 66.65, *p* = 1.72 × 10^−23^; Figure 1d). The mean Fv/Fm values of *V. vinifera* cultivars, interspecific hybrids and wild accessions were 0.238, 0.341 and 0.380, respectively. Games–Howell post hoc tests showed significant pairwise differences among all three groups.

Hierarchical clustering was performed using Fv/Fm at 47 °C as the input variable. The elbow plot showed a clear inflection at k = 3, supporting the division of the 404 accessions into three phenotypic clusters (Figure 1e). Cluster I contained 180 accessions and had a mean Fv/Fm of 0.231 ± 0.080. This cluster included 106 of the 158 *V. vinifera* cultivars, representing 67.1% of that group. Cluster II contained 101 accessions and had a mean Fv/Fm of 0.282 ± 0.101. Cluster III contained 123 accessions and had a mean Fv/Fm of 0.450 ± 0.072. This cluster included 55 of the 97 wild accessions, representing 56.7% of the wild group, as well as 56 interspecific hybrids (Figure 1f). Of the 21 candidate accessions with relatively high PSII thermal stability, 19 were assigned to Cluster III. Of the 21 candidate accessions with relatively low PSII thermal stability, 17 were assigned to Cluster I.

### 2.2. Genetic Structure Analysis

**Figure 2:**
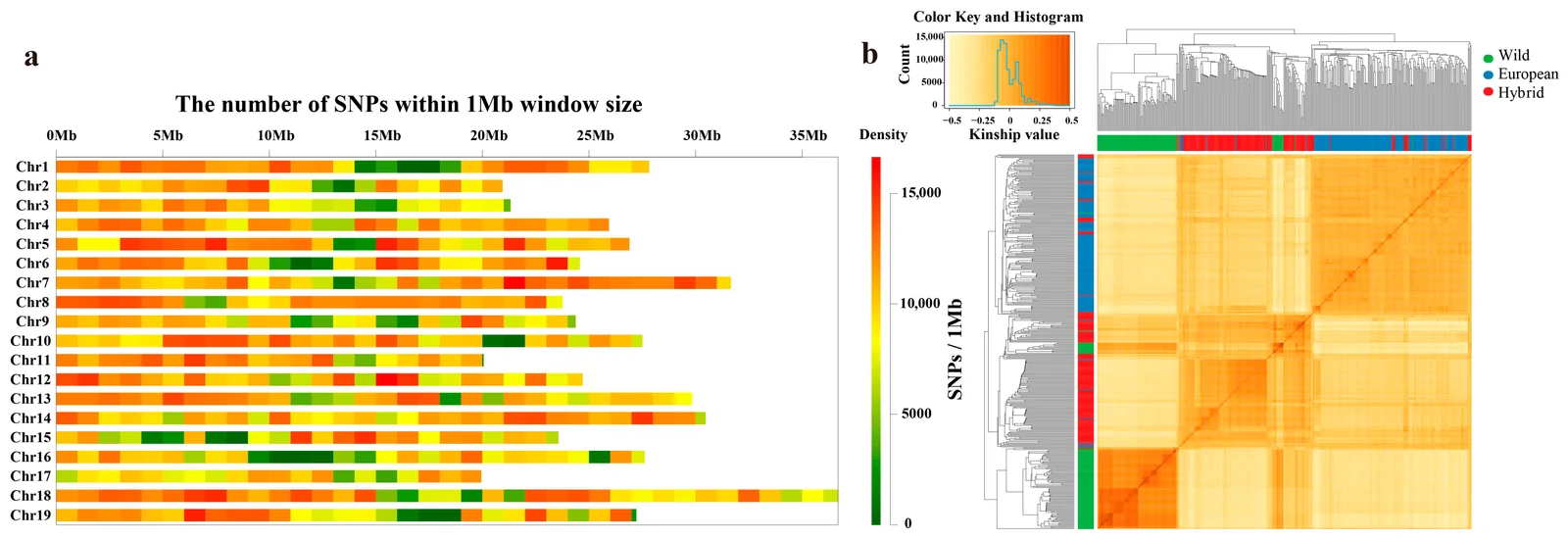
SNP density and kinship analyses. (**a**) Density and distribution of SNPs across the 19 grapevine chromosomes. Colours indicate marker density within 1 Mb windows, as shown by the scale on the right. (**b**) Heatmap of the kinship matrix for the 404 grapevine accessions. The histogram labelled “Kinship” shows the distribution of pairwise kinship coefficients across the matrix.

After filtering for a missing-data rate of ≤2% and a minor allele frequency (MAF) of ≥0.05, 5,074,860 high-quality SNPs were retained. These SNPs were distributed across the 19 chromosomes of the PN40024 grapevine reference genome (Figure 2a). SNP numbers varied among chromosomes, with Chr18 containing the largest number (390,077 SNPs). Most SNPs had MAF values between 0.05 and 0.30 (Supplementary Figure S1), accounting for 79.80% of all retained SNPs.

Individual heterozygosity across the 404 grapevine accessions ranged from 0.0405 to 0.5375. All values were below 0.6 and were concentrated mainly between 0.20 and 0.30 (Supplementary Figure S2). These results supported the use of this genotype dataset for subsequent GWAS. Kinship analysis further showed that pairwise relatedness was generally weak among the accessions (Figure 2b), consistent with the high genetic diversity of the association panel.

### 2.3. Genome-Wide Association Analysis

To identify genetic loci associated with variation in PSII thermal stability in grapevine, a genome-wide association study was performed using Fv/Fm measured after heat treatment as the phenotype. The analysis included 404 grapevine accessions and 5,074,860 high-quality SNPs and was conducted using a linear mixed model incorporating population structure and kinship (LMM + QK). Principal components and the kinship matrix were included to control for population structure and relatedness among accessions.

**Figure 3:**
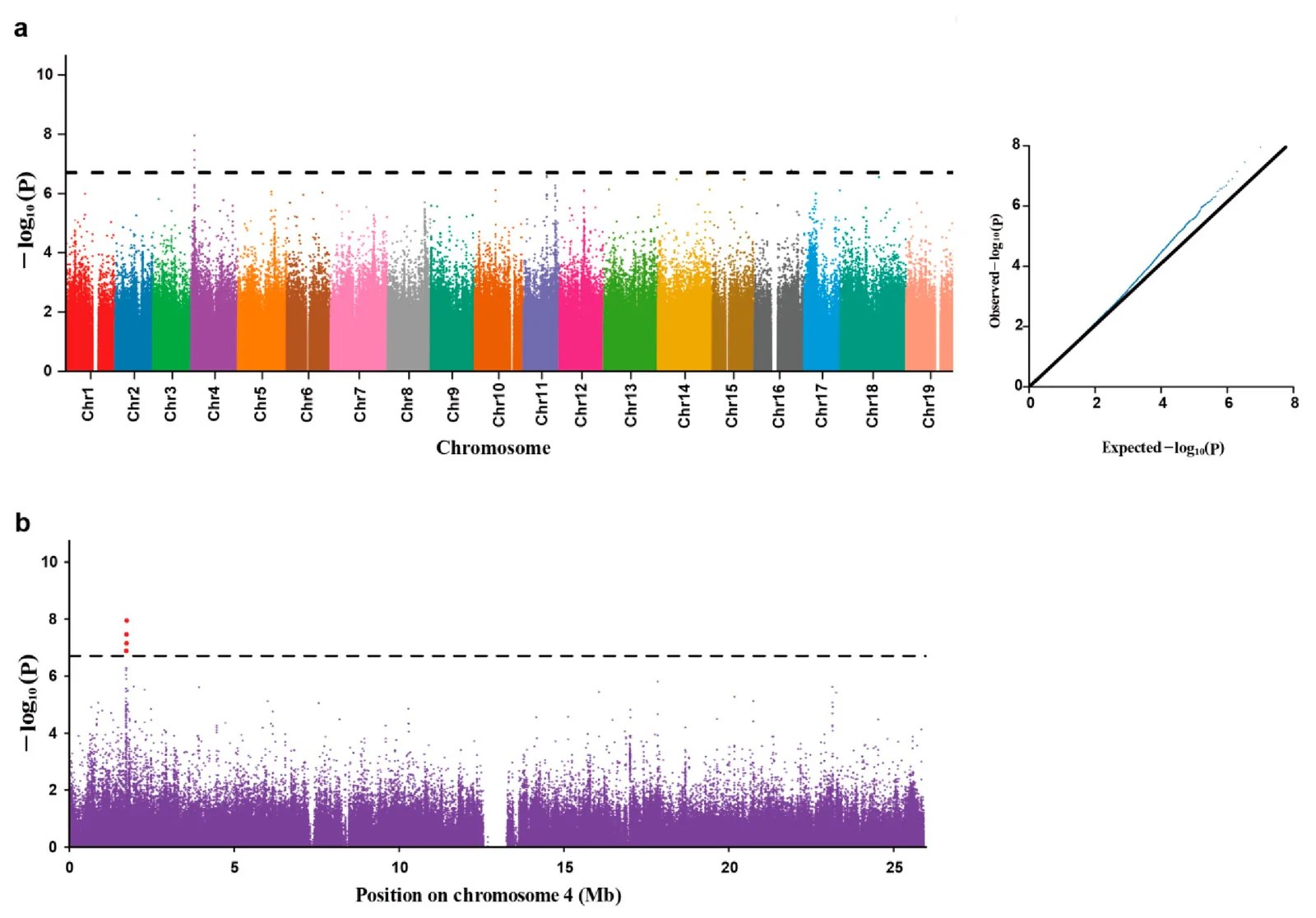
Genome-wide association analysis of Fv/Fm after heat treatment. (**a**) GWAS results for Fv/Fm after treatment at 47 °C. In the Manhattan plot (**left**), the x-axis shows chromosome number and the y-axis shows −log_10_(P). The black dashed line indicates the suggestive significance threshold of *p* = 1.97 × 10^−7^. In the Q-Q plot (**right**), the x- and y-axes show the expected and observed −log_10_(P) values, respectively. The black line represents the expected distribution under the null hypothesis, and the blue line represents the observed *p* values. (**b**) Regional association plot for chromosome 4. The x-axis shows chromosomal position, and the y-axis shows −log_10_(P). The black dashed line indicates the suggestive significance threshold of *p* = 1.97 × 10^−7^.

In the quantile–quantile (Q-Q) plot, the observed *p* values generally agreed with the expected distribution, indicating adequate control of potential confounding (Figure 3a). SNPs in the upper tail deviated markedly from the expected distribution, suggesting the presence of putative true associations. The Manhattan plot showed that the primary association signal for Fv/Fm after heat treatment was located on chromosome 4 (Chr4). The lead SNP, Chr4_1743280_G_A, had a *p* value of 2.58 × 10^−8^ and passed the suggestive significance threshold. Regional association analysis further identified a clear peak at approximately 1.7 Mb on Chr4, indicating that this region may contain candidate genes potentially associated with variation in PSII thermal stability (Figure 3b).

### 2.4. Prediction of Candidate Genes

A total of 44 genes were identified within the ±200 kb candidate interval surrounding the lead SNP Chr4_1743280_G_A (Supplementary Table S3). Based on functional annotations and published evidence related to heat responses or variation in Fv/Fm, nine genes were retained as preliminary candidates (Table 1). Subsequent variant annotation and diplotype–phenotype comparisons further prioritized seven genes as final candidates.

*Vitvi005402* was annotated as encoding an SMR domain-containing protein. The lead SNP was located within this gene, making it a strong positional candidate at the associated locus. *Vitvi005403* and *Vitvi005405* were annotated as encoding DYW domain-containing proteins and may participate in organellar RNA editing or processing. A stop-gained variant was identified in *Vitvi005405* and may cause premature termination of translation. *Vitvi005406* was annotated as encoding a pentatricopeptide repeat (PPR)-containing protein and also carried a stop-gained variant. This gene may be involved in organellar gene expression or heat-related stress responses.

*Vitvi005408* encoded an EF-hand domain-containing protein that may participate in Ca^2+^ signalling. *Vitvi005409* was annotated as encoding a BRCT domain-containing protein. Proteins containing this domain are commonly involved in DNA-damage repair and stress responses and may contribute to the repair of heat-induced damage. *Vitvi005412* encoded a PORR domain-containing protein. PORR domain proteins are often associated with organellar RNA metabolism and may indirectly affect chloroplast function. *Vitvi005414* encoded an AP2/ERF domain-containing protein, a typical class of stress-responsive transcription factors. *Vitvi005420* encoded a RING-type E3 ubiquitin ligase that may participate in ubiquitination and protein-homeostasis maintenance under heat stress.

Together, the candidate interval contained genes potentially involved in organellar RNA processing, DNA-damage repair, Ca^2+^ signalling, transcriptional regulation and protein homeostasis. These genes represent plausible candidates for natural variation in PSII thermal stability in grapevine, either individually or through coordinated functions.

### 2.5. Haplotype Analysis

After evaluating candidate regulatory and coding variants, seven genes were retained based on haplotype analysis and significant differences in Fv/Fm among major diplotype groups after heat treatment (Supplementary Table S4). Haplotypes were constructed from the selected variants in each candidate gene. The two phased haplotypes carried by each diploid accession were used to define its diplotype (Supplementary Table S5).

**Figure 4:**
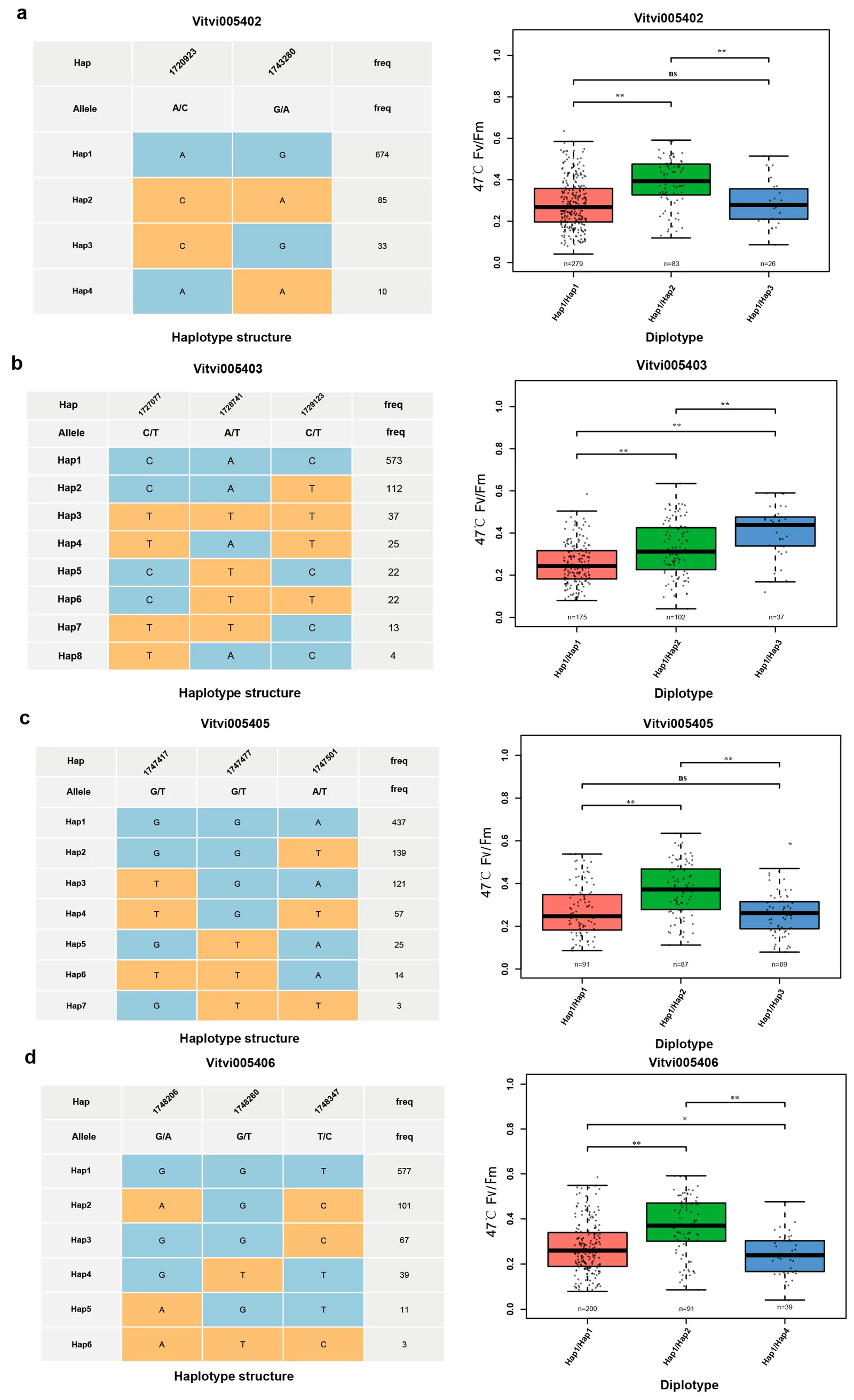

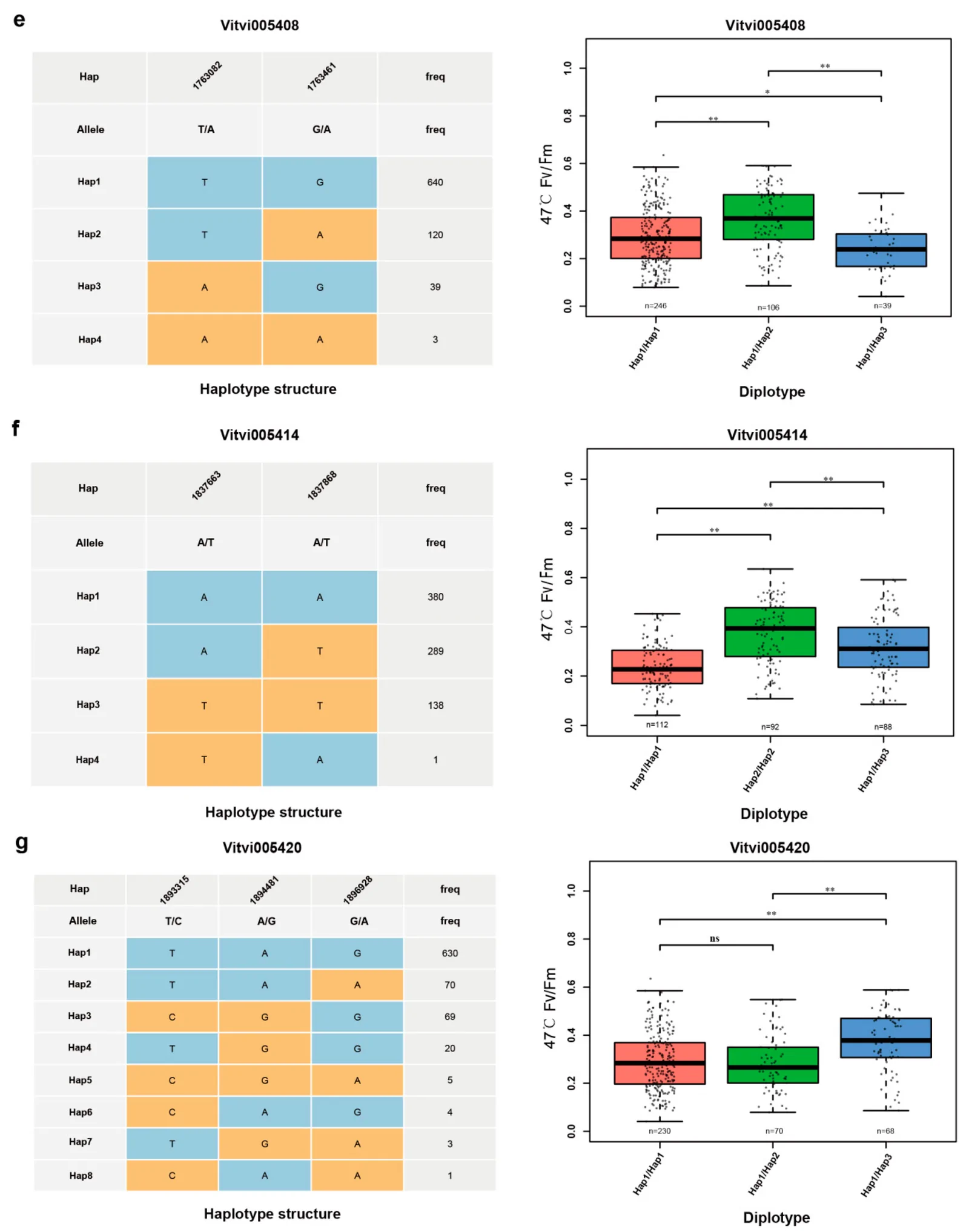
Haplotype and diplotype analyses of seven candidate genes. Haplotype diagrams show the allele combinations of selected SNPs in (**a**) *Vitvi005402*, (**b**) *Vitvi005403*, (**c**) *Vitvi005405*, (**d**) *Vitvi005406*, (**e**) *Vitvi005408*, (**f**) *Vitvi005414* and (**g**) *Vitvi005420*. Each column represents a specific SNP, with its genomic coordinate shown at the top. The letters A, T, C and G indicate the nucleotide present at each site. For allele labels such as A/G, the first letter denotes the reference allele in the PN40024 reference genome, and the second denotes the alternative allele observed in the population. Each coloured cell indicates the allele carried by a haplotype at the corresponding SNP. The “Freq” column indicates the observed number of haplotype copies among the 404 diploid accessions. Different allele combinations define the haplotypes used to analyse Fv/Fm after treatment at 47 °C. In each panel, haplotype information is shown on the left, and pairwise comparisons among the three most frequent diplotype groups are shown on the right. Comparisons were performed using Welch’s two-sample *t*-test, with *p* values adjusted using the Benjamini–Hochberg method. The horizontal line within each box indicates the median. * indicates *p* < 0.05, ** indicates *p* < 0.01 and ns indicates no significant difference.

*Vitvi005402* was annotated as encoding an SMR domain-containing protein. Two candidate SNPs were selected within this gene. Chr4_1720923_A_C was a nonsynonymous variant in the coding sequence (CDS) that resulted in a Glu99Ala substitution. Chr4_1743280_G_A was located in an intron. Four observed haplotypes were defined by these SNPs. Hap1 (A-G) was the most frequent, whereas the remaining haplotypes were Hap2 (C-A), Hap3 (C-G) and Hap4 (A-A). At 47 °C, accessions carrying Hap1/Hap2 had significantly higher Fv/Fm than those carrying Hap1/Hap1 or Hap1/Hap3. No significant difference was detected between Hap1/Hap1 and Hap1/Hap3 (Figure 4a).

*Vitvi005403* was annotated as encoding a DYW domain-containing protein. Three nonsynonymous SNPs were identified in its CDS. Chr4_1727077_C_T resulted in a Pro15Leu substitution, Chr4_1728741_A_T resulted in an Ile570Phe substitution and Chr4_1729123_C_T resulted in an Ala697Val substitution. These variants defined eight observed haplotypes, of which Hap1 (C-A-C) and Hap2 (C-A-T) were the most frequent. Hap1/Hap1, Hap1/Hap2 and Hap1/Hap3 were the three most frequent diplotypes. Accessions carrying Hap1/Hap2 or Hap1/Hap3 had significantly higher Fv/Fm at 47 °C than those carrying Hap1/Hap1. Fv/Fm was also significantly higher in Hap1/Hap3 than in Hap1/Hap2 accessions (Figure 4b).

*Vitvi005405* was also annotated as encoding a DYW domain-containing protein. Three SNPs were identified in its CDS, comprising two nonsynonymous variants and one stop-gained variant. Chr4_1747417_G_T resulted in an Asp323Tyr substitution, and Chr4_1747501_A_T resulted in an Ile351Phe substitution. Chr4_1747477_G_T converted Glu343 into a stop codon, resulting in premature termination. These variants defined seven observed haplotypes. Hap1 (G-G-A), Hap2 (G-G-T) and Hap3 (T-G-A) were the major haplotypes. Accessions carrying Hap1/Hap2 had significantly higher Fv/Fm at 47 °C than those carrying Hap1/Hap1 or Hap1/Hap3. No significant difference was detected between Hap1/Hap1 and Hap1/Hap3 (Figure 4c).

*Vitvi005406* was annotated as encoding a pentatricopeptide repeat (PPR)-containing protein. Three SNPs were identified in its CDS, including two nonsynonymous variants and one stop-gained variant. Chr4_1748206_G_A resulted in a Val108Ile substitution, whereas Chr4_1748347_T_C resulted in a Phe155Leu substitution. Chr4_1748260_G_T converted Gly126 into a stop codon, resulting in premature termination. Six observed haplotypes were defined by these variants. Hap1 (G-G-T) was the predominant haplotype, whereas Hap2 (A-G-C) and Hap3 (G-G-C) were the major secondary haplotypes. Accessions carrying Hap1/Hap2 had significantly higher Fv/Fm at 47 °C than those carrying Hap1/Hap1 or Hap1/Hap4. The Hap1/Hap4 group showed relatively low Fv/Fm (Figure 4d).

*Vitvi005408* was annotated as encoding an EF-hand domain-containing protein. Two candidate SNPs, Chr4_1763082_T_A and Chr4_1763461_G_A, were selected from its promoter region. These SNPs defined four observed haplotypes. Hap1 (T-G) was the predominant haplotype, whereas Hap2 (T-A) and Hap3 (A-G) were the major secondary haplotypes. Accessions carrying Hap1/Hap2 had significantly higher Fv/Fm at 47 °C than those carrying Hap1/Hap1 or Hap1/Hap3. The Hap1/Hap3 group showed relatively low Fv/Fm (Figure 4e).

*Vitvi005414* was annotated as encoding an AP2/ERF domain-containing protein. Two nonsynonymous SNPs were identified in its CDS. Chr4_1837663_A_T resulted in a Phe187Tyr substitution, whereas Chr4_1837868_A_T resulted in a Ser119Thr substitution. These SNPs defined four observed haplotypes, of which Hap1 (A-A), Hap2 (A-T) and Hap3 (T-T) were the most frequent. Accessions carrying Hap2/Hap2 had significantly higher Fv/Fm at 47 °C than those carrying Hap1/Hap1 or Hap1/Hap3. Fv/Fm was also significantly higher in Hap1/Hap3 than in Hap1/Hap1 accessions (Figure 4f).

*Vitvi005420* was annotated as encoding a RING-type E3 ubiquitin ligase. Three candidate SNPs were selected within or near this gene. Chr4_1893315_T_C was a nonsynonymous variant in the CDS that resulted in a Thr258Ala substitution. Chr4_1894481_A_G was located in an intron, and Chr4_1896928_G_A was located in the 2 kb upstream promoter region. These variants defined eight observed haplotypes. Hap1 (T-A-G) was the predominant haplotype, whereas Hap2 (T-A-A) and Hap3 (C-G-G) were the major secondary haplotypes. Accessions carrying Hap1/Hap3 had significantly higher Fv/Fm at 47 °C than those carrying Hap1/Hap1 or Hap1/Hap2. No significant difference was detected between Hap1/Hap1 and Hap1/Hap2 (Figure 4g).

## 3. Discussion

### 3.1. Phenotypic Evaluation of PSII Thermal Stability in Grapevine Germplasm

We evaluated PSII thermal stability in 404 genetically diverse grapevine accessions using a standardized treatment at 47 °C. Heat treatment markedly increased variation in Fv/Fm, allowing accessions with contrasting PSII thermal stability to be distinguished. Previous work showed that short-term treatment at 47 °C combined with chlorophyll fluorescence measurements sensitively detected heat injury in grapevine leaves [6]. Another study combined Fv/Fm with a heat-injury index and electrolyte leakage to assess heat tolerance across grapevine accessions [7]. Together, these findings support Fv/Fm as a practical indicator for the initial screening of PSII thermal stability in large grapevine panels. The 47 °C, 40 min treatment was a standardized acute assay for comparative screening of PSII thermal stability rather than a simulation of field heat exposure; field performance therefore requires further validation. More broadly, genomic analyses of arugula germplasm illustrate how systematic germplasm characterization can reveal genetic diversity and identify valuable breeding resources [24].

Previous surveys have revealed substantial variation in heat responses among grapevine accessions. Large-scale screening has focused mainly on Chinese wild grapevine resources [8]. Heat-tolerance classifications have also been developed using smaller panels comprising cultivars, interspecific hybrids and wild accessions [9]. Our larger panel included *Vitis vinifera* cultivars, interspecific hybrids and diverse wild accessions. Wild accessions generally retained higher Fv/Fm after treatment at 47 °C, whereas *V. vinifera* cultivars showed lower values overall. All accessions in the top 5% of the phenotypic distribution were either wild accessions or interspecific hybrids. These findings indicate that wild germplasm and some interspecific hybrids may provide valuable genetic variation associated with enhanced PSII thermal stability and may serve as useful resources for future heat-resilience breeding.

Nevertheless, the three germplasm groups overlapped across the phenotypic clusters. Group-level differences therefore cannot be used to infer the broader heat tolerance of individual accessions from PSII thermal stability alone. Selection should remain based on directly measured phenotypes for each accession. The candidate accessions with relatively high and low PSII thermal stability identified here provide representative materials for physiological comparisons, genetic population development and molecular-marker validation. However, the present phenotypic evaluation was conducted in a single year, at one location, and at a single sampling time, and was based mainly on leaf Fv/Fm following short-term heat treatment. Therefore, multi-year and multi-environment evaluations with repeated measurements, together with whole-vine experiments, will be required to determine the stability and broader relevance of the observed phenotypic differences among these candidate accessions.

### 3.2. Differences in Fv/Fm Among Diplotype Groups of Seven Candidate Genes

We identified 18 putatively functional SNPs across the seven prioritized candidate genes. These included 11 nonsynonymous variants, two stop-gained variants, three promoter variants and two intronic variants. These variant classes may affect protein structure or function, gene transcription or RNA processing. Previous studies have linked natural variation in the coding and promoter regions of grapevine *HSFA2*, as well as an intronic variant affecting the transcriptional regulation of *TTC4*, to differences in heat tolerance [14,23]. These findings indicate that natural variants in different genic regions may contribute to heat responses in grapevine.

Under the predefined diplotype-screening criteria, all seven retained genes showed a significant difference in Fv/Fm after treatment at 47 °C in at least one comparison among the major diplotypes. Higher Fv/Fm was associated with Hap1/Hap2 in *Vitvi005402*, *Vitvi005405*, *Vitvi005406* and *Vitvi005408*; with Hap1/Hap3 in *Vitvi005403* and *Vitvi005420*; and with Hap2/Hap2 in *Vitvi005414*. Except for *Vitvi005414*, the higher Fv/Fm values were observed mainly in heterozygous diplotypes. This pattern could reflect dominance, heterozygote-specific effects or interactions with the local genetic background. However, the present data cannot distinguish among these explanations or establish that Hap2 or Hap3 alone has a stable favourable effect.

By contrast, Hap2/Hap2 of *Vitvi005414* was homozygous and showed significantly higher Fv/Fm than Hap1/Hap1 and Hap1/Hap3. Hap2 may therefore be prioritized for further validation. However, diplotype differences were used only to prioritize and rank candidate genes. They do not provide independent evidence for a causal effect of any candidate gene or individual variant.

### 3.3. Potential Roles of Candidate Genes in PSII Thermal Stability and Heat Responses

Integration of genomic position, functional annotation, putatively functional variants and diplotype–phenotype differences grouped the seven candidate genes into three putative functional categories. *Vitvi005402*, *Vitvi005403*, *Vitvi005405* and *Vitvi005406* may contribute to organellar RNA processing and the maintenance of chloroplast function. *Vitvi005408* and *Vitvi005414* may participate in Ca^2+^ signalling and stress-responsive transcriptional regulation, respectively. *Vitvi005420* may contribute to ubiquitin-mediated protein quality control.

*Vitvi005414* encodes an AP2/ERF domain-containing transcription factor. The AP2/ERF family is widely involved in plant responses to abiotic stress. Arabidopsis ERF1 responds to multiple stress signals and regulates downstream gene expression [25]. Rice *OsERF115*/*AP2EREBP110* is induced by heat and drought, and its overexpression enhances tolerance to both stresses [26]. We identified two nonsynonymous variants in *Vitvi005414*, p.Ser119Thr and p.Phe187Tyr. Accessions carrying the Hap2/Hap2 diplotype also had higher Fv/Fm after treatment at 47 °C. In contrast to the other candidate genes, for which higher Fv/Fm was associated mainly with heterozygous diplotypes, the association with homozygous Hap2/Hap2 provides a clearer basis for prioritizing Hap2. *Vitvi005414* is therefore a strong candidate for marker validation in independent populations, expression analysis and functional characterization.

*Vitvi005406* is annotated as encoding a pentatricopeptide repeat (PPR) protein. PPR proteins are key regulators of organellar RNA metabolism and participate in RNA editing, splicing, stabilization and translation [27]. The Arabidopsis PPR protein RARE1 mediates RNA editing of the chloroplast *accD* transcript. Loss of RARE1 disrupts fatty-acid synthesis and membrane-lipid homeostasis and increases sensitivity to heat [28]. We identified two nonsynonymous variants, p.Val108Ile and p.Phe155Leu, and a stop-gained variant, p.Gly126Ter (G126*), in *Vitvi005406*. Because G126* occurs relatively early in the protein sequence, it could cause extensive C-terminal truncation and potentially affect RNA recognition or processing. Together with the observed Fv/Fm differences among major diplotypes, these findings prioritize *Vitvi005406* and G126* for further investigation. However, the proposed involvement of *Vitvi005406* in organellar RNA processing and heat responses remains putative because direct functional evidence for this gene in grapevine is currently lacking.

*Vitvi005402*, *Vitvi005403* and *Vitvi005405* may also contribute to organellar RNA processing and the maintenance of chloroplast function. Several RNA-editing sites in grapevine chloroplasts and mitochondria respond to changes in temperature, indicating a possible role for organellar RNA processing in temperature adaptation [29]. *Vitvi005402* encodes an SMR domain-containing protein and contains the lead SNP, providing strong positional evidence. The rice and maize PPR-SMR protein ATP4 mediates RNA editing of the chloroplast *rps8* transcript and affects chloroplast RNA accumulation and protein synthesis [30]. The Arabidopsis PPR-SMR protein GUN1 contributes to plastid-to-nucleus signalling and basal heat tolerance [31]. *Vitvi005403* and *Vitvi005405* are annotated as encoding DYW domain-containing proteins. The DYW domain can catalyse C-to-U RNA editing [32]. Loss of rice *OsDYW2* disrupts chloroplast RNA editing and splicing under heat stress and causes defects in chloroplast development [33]. *Vitvi005405* also carries the stop-gained variant p.Glu343Ter, which is predicted to truncate the protein. However, these proposed functions are inferred primarily from domain annotations and studies of homologous proteins in other plant species rather than from direct functional evidence in grapevine. Therefore, whether *Vitvi005403* and *Vitvi005405* affect organellar RNA editing, splicing or translation in grapevine remains to be experimentally validated.

Functional evidence for *Vitvi005408* and *Vitvi005420* is currently more limited. *Vitvi005408* encodes an EF-hand domain-containing protein and may participate in Ca^2+^ sensing or organellar Ca^2+^ homeostasis. *Arabidopsis AtCaM3* participates in heat-stress signalling, and its loss reduces both basal and acquired heat tolerance [34]. The EF-hand protein MICU regulates mitochondrial Ca^2+^ dynamics [35]. *Vitvi005420* encodes a RING-type E3 ubiquitin ligase and may contribute to protein quality control and stress signalling under high temperature. The rice E3 ligases OsHTAS and OsHIRP1 positively regulate heat responses [36,37], whereas Arabidopsis MPSR1 recognizes and removes newly synthesized misfolded proteins [38]. These studies provide indirect support for the proposed functions of *Vitvi005408* and *Vitvi005420*, but they do not establish equivalent roles in grapevine.

Overall, the Hap2/Hap2 diplotype of *Vitvi005414* and the G126* stop-gained variant in *Vitvi005406* warrant high priority for validation. *Vitvi005402* and *Vitvi005405* are also supported by strong positional evidence and a predicted protein-truncating variant, respectively. However, diplotype differences provide a basis for candidate prioritization rather than independent evidence of causality. Likewise, the functions of homologous proteins in other plant species cannot establish equivalent functions for the corresponding grapevine genes. An important limitation of the present study is that the candidate genes have not yet been functionally validated using genetic transformation or genome-editing approaches in grapevine. Future work should therefore combine marker validation in independent populations, heat-induced expression profiling, assays of organellar RNA processing and protein integrity, and gene overexpression or genome-editing experiments. These approaches will be required to determine the contributions of the candidate genes and key variants to PSII thermal stability in grapevine.

## 4. Materials and Methods

### 4.1. Plant Materials and Growth Conditions

A total of 404 grapevine accessions were used for the association analysis, including 158 *V. vinifera* cultivars, 149 interspecific hybrids and 97 wild accessions. All plant materials were obtained from the National Grape Germplasm Repository at the Zhengzhou Fruit Research Institute, Chinese Academy of Agricultural Sciences, Zhengzhou, China (34°48′ N, 113°42′ E). The experimental site has a mean annual temperature of 14.2 °C, annual precipitation of 666.0 mm, 2436 h of annual sunshine and a frost-free period of 213 days. The soil is brown sandy loam with a pH of 7.1–7.5. The vines were trained to a Y-shaped system with a single trunk and two cordons. Vine and row spacing were 1.0 m and 2.5 m, respectively, and the trellis height was 1.8 m. All accessions were grown under open-field conditions and were managed under the same field-management regime, including standardized fertilization.

### 4.2. Measurement of Fv/Fm

The experiment was conducted in May 2026 to evaluate variation in PSII thermal stability among grapevine accessions. At the time of sampling, the vines were at the pre-flowering stage under local field conditions. Leaves were collected between 08:00 and 10:00 to minimize variation associated with leaf developmental stage and diurnal changes in chlorophyll fluorescence. For each accession, three vines with similar growth vigour were randomly selected, with each vine serving as one biological replicate. One mature functional leaf was collected from each vine. Leaves were sampled from nodes 6–8 of current-season shoots. They were approximately 30 days old, fully expanded, exposed to similar light conditions and free from disease, insect damage and mechanical injury. Thus, three biological replicates were included for each accession.

The leaves were immediately placed in sealed bags, and leaf discs were prepared within 30 min of sampling. Two discs, each 5.5 cm in diameter, were excised from similar positions on each leaf while avoiding the main vein and major lateral veins. The two discs from the same leaf were randomly assigned to the 25 °C control or the 47 °C heat treatment. Each temperature treatment therefore included three biological replicates. This paired design minimized variation among individual leaves. Each vine was considered an independent biological replicate, and the two leaf discs derived from the same leaf constituted a matched pair for the two temperature treatments.

The discs were placed on moist filter paper in aluminium boxes to prevent water loss and exclude external light. The boxes were then transferred to water baths maintained at 25 °C or 47 °C, and the discs were treated in darkness for 40 min [6]. This treatment procedure, including the use of moist filter paper, aluminium containers and a temperature-controlled water bath, followed established grapevine leaf-disc heat-treatment protocols [6,23]. The 47 °C treatment for 40 min was therefore selected as a standardized acute heat-treatment regime for comparative screening of PSII thermal stability across the large germplasm panel, rather than as a direct simulation of field heat exposure. The leaf discs did not directly contact the water during treatment.

Immediately after treatment, fast chlorophyll fluorescence induction kinetics were measured using a portable Plant Efficiency Analyser (Handy PEA; Hansatech Instruments Ltd., King’s Lynn, UK). Minimum fluorescence (F_0_) and maximum fluorescence (F_m_) were recorded. The maximum quantum efficiency of photosystem II (PSII) was calculated as follows:

F_v_/F_m_ = (F_m_ − F_0_)/F_m_

The 40 min temperature treatment in darkness also served as the dark-adaptation period. Three biological replicate measurements were obtained for each accession under both temperature conditions, and the mean was calculated separately for each treatment. The mean F_v_/F_m_ of the three biological replicates after treatment at 47 °C was used as the phenotype for GWAS to investigate genetic variation in PSII maximum quantum efficiency under heat stress.

### 4.3. Genome Sequencing and Reference Genome Information

Young leaves were collected from 404 grapevine accessions, immediately snap-frozen in liquid nitrogen, and stored at −80 °C until DNA extraction. Total genomic DNA was extracted by Annoroad Gene Technology Co., Ltd. (Beijing, China). Paired-end DNA libraries with an average insert size of approximately 350 bp were constructed and sequenced on an Illumina platform using a 150 bp paired-end strategy. Each accession was sequenced to an average depth of 30×, and the resulting data were used for population-level SNP discovery.

Raw sequencing data were subjected to stringent quality control. High-quality clean reads were obtained by removing: (1) reads with more than 5 bp overlapping an adapter sequence; (2) low-quality reads in which more than 50% of bases had a quality score below 19; and (3) reads containing more than 5% undetermined bases (N). Variants were further filtered using a minor allele frequency (MAF) threshold of ≥0.05 and a missing-data rate of ≤2%. After filtering, 5,074,860 SNPs were retained across the 404 accessions for subsequent analyses.

The grape PN40024 V6 genome was used as the reference genome [39]. The reference genome sequence and annotation files were obtained from https://zenodo.org/records/10969867 (accessed on 6 August 2026).

### 4.4. Genotyping and Genetic Structure Analysis

PLINK (version 1.90b6.18) was used to estimate heterozygosity for each accession and the minor allele frequency (MAF) of each SNP across the population. TASSEL (version 5.2.89) [40] was used for kinship analysis, and the kinship matrix and SNP density plot were generated. Data visualization was performed using packages in R (version 4.5.0).

### 4.5. GWAS Analysis

Genome-wide marker-trait associations were tested using the univariate linear mixed model implemented in GEMMA (genome-wide efficient mixed-model association, version 0.98.3) [17]. Population-structure covariates and a genomic kinship matrix were included to control for genetic stratification and relatedness among accessions. The model was expressed as:

y = Wα + xβ + u + ε

where y is the phenotype vector; W is the covariate matrix containing an intercept and the selected principal components; α is the corresponding vector of fixed effects; x is the genotype vector of the tested SNP; β is the fixed effect of the tested SNP; u is the polygenic random-effect vector; and ε is the residual-error vector. The covariance structure of u was determined by the genomic kinship matrix K.

Population-structure covariates were obtained through principal component analysis of the genotype matrix using PLINK (version 1.90b6.18). The first three principal components (PC1-PC3) were included in the association model because they explained 24.32%, 18.02%, and 8.86% of the genetic variation, respectively, accounting for 51.20% in total, and captured the major axes of population structure among the *V. vinifera* cultivars, interspecific hybrids, and wild accessions. Bonferroni correction was based on all 5,074,860 retained SNPs. This gave a Bonferroni threshold of 0.05/m = 9.85 × 10^−9^ and a suggestive threshold of 1/m = 1.97 × 10^−7^, where m is the total number of retained SNPs. The suggestive threshold was used to retain moderate-effect association signals for subsequent candidate-gene screening. Manhattan and quantile–quantile (Q-Q) plots were generated using CMplot (version 4.5.1) in R.

### 4.6. Identification of Potential Candidate Genes

For each lead SNP, the initial candidate interval was defined as 200 kb upstream and downstream of the SNP. Linkage disequilibrium decay in grapevine varies substantially among genetic groups and genomic regions, ranging from a few kilobases to several hundred kilobases [41]. Therefore, the ±200 kb interval was used as a pragmatic initial screening window to capture genes located near the association signal while avoiding an excessively narrow search region. This interval was not intended to represent a strict LD-defined confidence interval; candidate genes within the window were subsequently prioritized based on functional annotation, variant type, and diplotype–phenotype comparisons.

Candidate genes were prioritized using a three-stage screening procedure. First, all genes within the ±200 kb candidate interval were extracted from the reference genome annotation, yielding 44 genes. Based on functional annotations and published evidence, genes potentially involved in heat responses or variation in Fv/Fm were retained as preliminary candidates, reducing the candidate set from 44 to nine genes. Second, variants within the nine preliminary candidate genes and their flanking regions (2 kb upstream and 1 kb downstream) were functionally annotated using ANNOVAR (version 2025Mar02) [42]. Nonsynonymous coding variants, stop-gained variants and putative regulatory variants were prioritized for haplotype construction in R. The two phased haplotypes carried by each diploid accession were used to define its diplotype. Third, Fv/Fm was compared among the major diplotype groups of each preliminary candidate gene. Genes showing a significant difference in Fv/Fm (*p* < 0.05) in at least one comparison among major diplotype groups were retained as final candidate genes. Based on these criteria, seven of the nine preliminary candidate genes were retained for subsequent analysis and interpretation.

### 4.7. Statistical Analyses

Phenotypic records were organized in Microsoft Excel 2016 (version 16.0). Data are presented as the mean ± standard deviation (SD). Statistical analyses were performed using R 4.6.1 and Origin 2024. Normality was assessed using the Shapiro–Wilk test and inspection of Q–Q plots. Although minor departures from normality were detected in some groups, Q–Q plots did not indicate severe deviations from normality. Differences among germplasm groups were evaluated using Welch’s one-way analysis of variance (ANOVA), followed by the Games–Howell post hoc test. Pairwise comparisons between the 25 °C and 47 °C treatments were performed using a paired-samples t-test. Pairwise comparisons among major diplotype groups were performed using Welch’s two-sample t-test, with *p* values adjusted using the Benjamini–Hochberg method. Differences were considered statistically significant at *p* < 0.05, or adjusted *p* < 0.05 where multiple-testing correction was applied [43]. Figures were generated using Origin, R and GraphPad Prism (version 11.0.1).

## 5. Conclusions

This study evaluated PSII thermal stability in 404 diverse grapevine accessions using Fv/Fm after treatment at 47 °C and performed GWAS based on 5,074,860 high-quality SNPs. Heat treatment revealed substantial phenotypic variation, with wild accessions generally retaining higher Fv/Fm than *V. vinifera* cultivars. Based on the phenotypic distribution, 21 accessions in the upper 5% and 21 accessions in the lower 5% were identified as candidate germplasm with relatively high and low PSII thermal stability, respectively. GWAS identified a suggestive association region on chromosome 4. Integration of the association signal, functional annotations, putatively functional variants and diplotype–phenotype comparisons prioritized seven candidate genes: *Vitvi005402*, *Vitvi005403*, *Vitvi005405*, *Vitvi005406*, *Vitvi005408*, *Vitvi005414* and *Vitvi005420*. Significant differences in Fv/Fm after treatment at 47 °C were detected among major diplotype groups for all seven genes, with the Hap2/Hap2 diplotype of *Vitvi005414* showing higher Fv/Fm. These candidate genes and diplotype associations should be interpreted as hypotheses for further validation rather than as evidence of causal effects. These findings provide candidate germplasm and genetic targets for further validation and molecular-marker development. Multi-year and multi-environment phenotyping, validation in independent populations and functional experiments will be required to confirm the stability and biological effects of the identified candidates.

## Figures and Tables

**Table 1:** Candidate genes prioritized for PSII thermal stability within the chromosome 4 GWAS region.

Chr	Peak SNP	Allele	Gene ID	Gene Start (bp)	Gene End (bp)	Gene Annotation
4	Chr4_1743280_G_A	G/A	*Vitvi005402*	1,719,159	1,745,183	SMR domain-containing protein
4	Chr4_1743280_G_A	G/A	*Vitvi005403*	1,727,034	1,730,042	DYW domain-containing protein
4	Chr4_1743280_G_A	G/A	*Vitvi005405*	1,746,451	1,747,662	DYW domain-containing protein
4	Chr4_1743280_G_A	G/A	*Vitvi005406*	1,747,690	1,749,843	Pentatricopeptide repeat-containing protein
4	Chr4_1743280_G_A	G/A	*Vitvi005408*	1,764,256	1,765,962	EF-hand domain-containing protein
4	Chr4_1743280_G_A	G/A	*Vitvi005409*	1,789,570	1,805,558	BRCT domain-containing protein
4	Chr4_1743280_G_A	G/A	*Vitvi005412*	1,821,840	1,825,745	PORR domain-containing protein
4	Chr4_1743280_G_A	G/A	*Vitvi005414*	1,837,391	1,838,278	AP2/ERF domain-containing protein
4	Chr4_1743280_G_A	G/A	*Vitvi005420*	1,892,711	1,895,344	RING-type E3 ubiquitin transferase

## Data Availability

The raw data supporting the conclusions of this article will be made available by the authors on reasonable request.

## Supplementary Materials

The following supporting information can be downloaded at https://www.mdpi.com/article/10.3390/ijms27209214/s1.
