# Supplementary material for: Evaluation of PSII Thermal Stability in Grapevine Germplasm and Identification of Candidate Genes Based on a Genome-Wide Association Study
Source: Int J Mol Sci. 2026 Oct 17;27(20):9214. doi: 10.3390/ijms27209214 (PMC13705109; doi:10.3390/ijms27209214)
Supplement: Supplementary file 1 [file ijms-27-09214-s001.zip › Supplementary Figures.pdf]

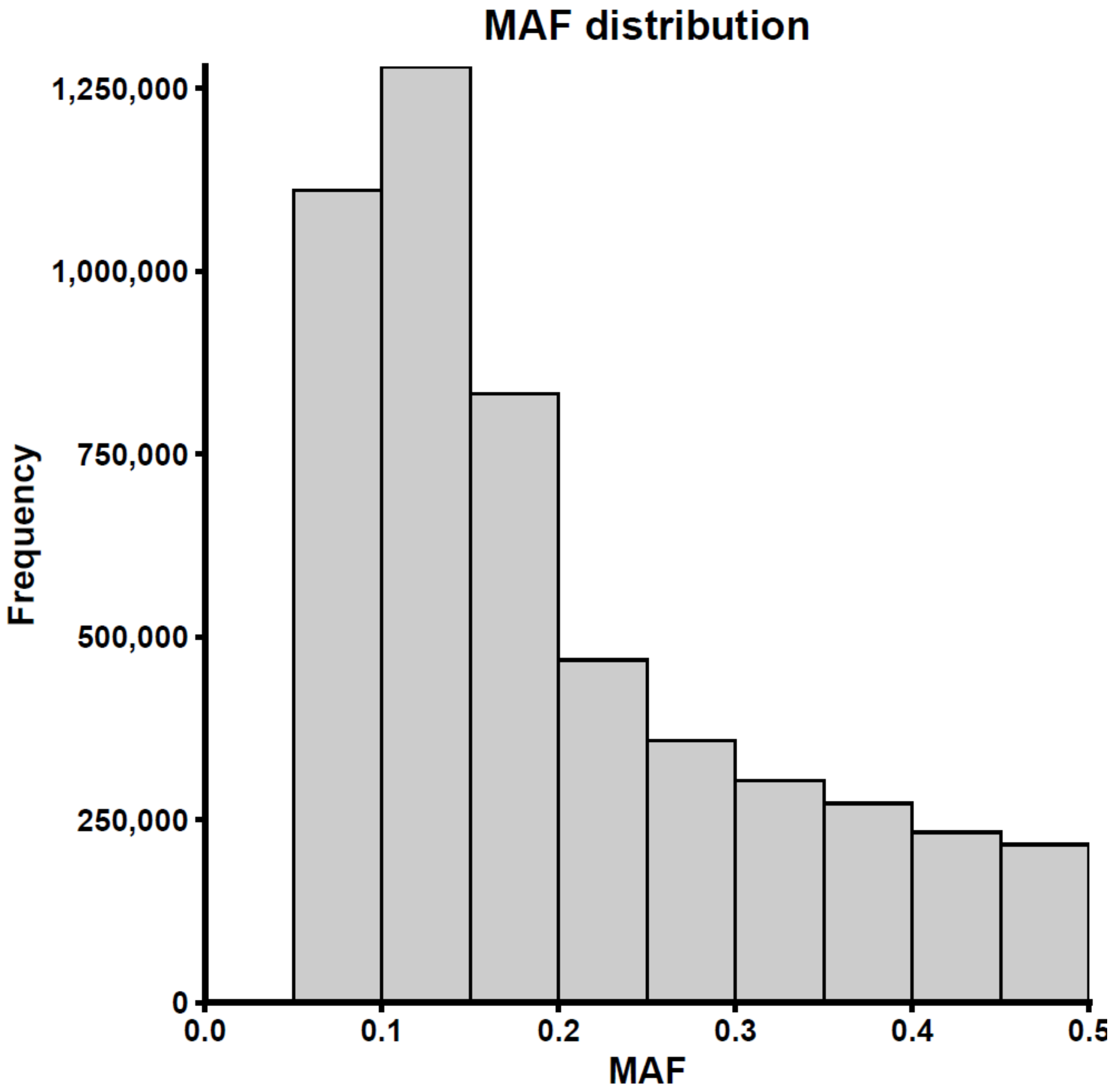

Figure S1. Distribution of the minor allele frequency (MAF) of 5,074,860 high-quality SNPs.

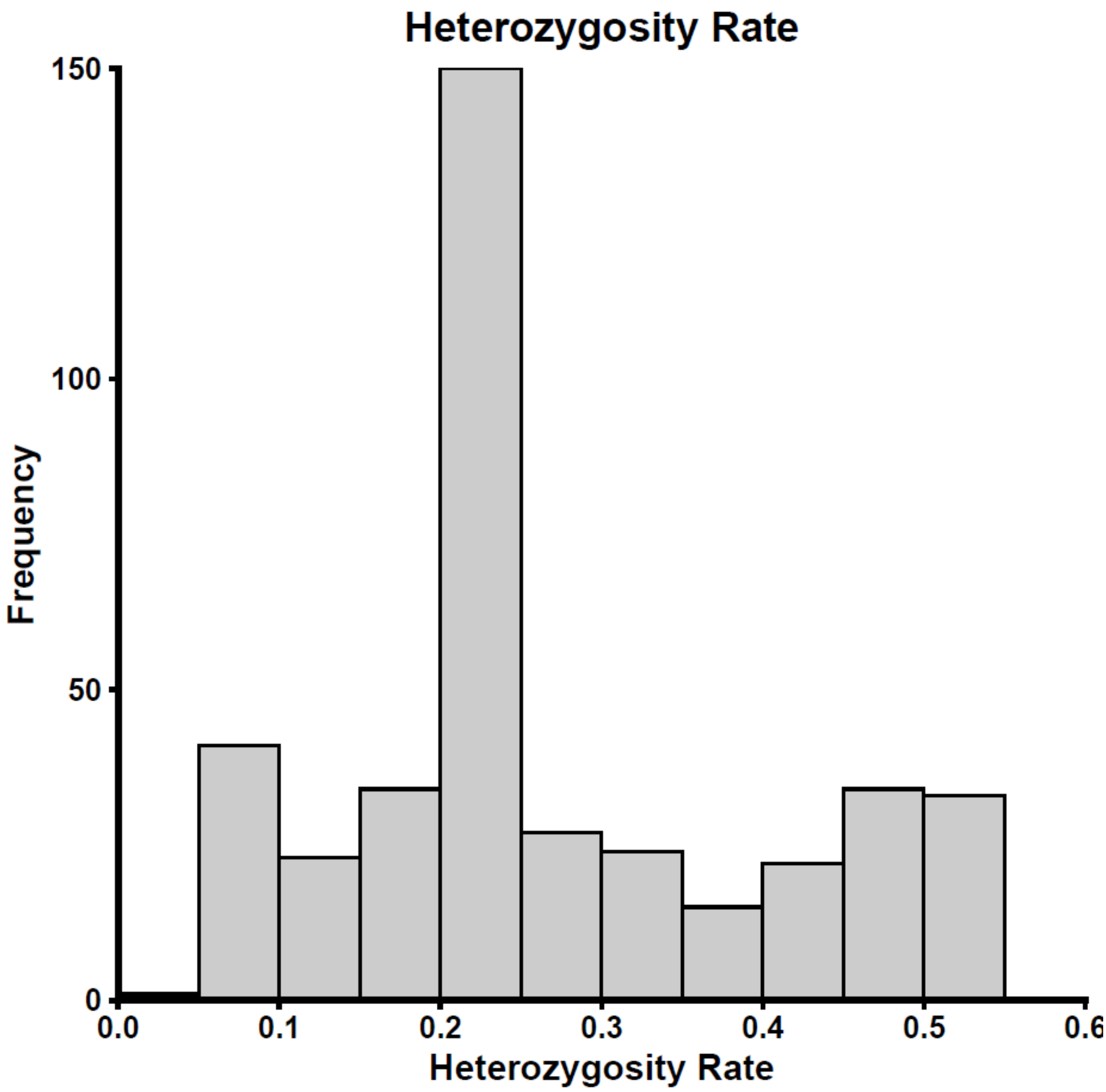

Figure S2. Distribution of individual heterozygosity rates among 404 grapevine accessions.
